# Loss of neuronal βPix isoforms impairs neuronal morphology in the hippocampus and causes behavioral defects

**DOI:** 10.1080/19768354.2024.2448999

**Published:** 2025-01-08

**Authors:** Younghee Kwon, Seung Joon Lee, Yoon Kyung Shin, June-Seek Choi, Dongeun Park, Jung Eun Shin

**Affiliations:** a School of Biological Sciences, Seoul National University, Seoul, Republic of Korea; b Department of Molecular Neuroscience, College of Medicine, Dong-A University, Busan, Republic of Korea; c Department of Psychology, Korea University, Seoul, Republic of Korea; d Department of Translational Biomedical Sciences, Graduate School of Dong-A University, Busan, Republic of Korea

**Keywords:** Arhgef7, guanine nucleotide exchange factor, dendritic spine, synaptic density

## Abstract

βPix is a guanine nucleotide exchange factor for the Rac1 and Cdc42 small GTPases, which play important roles in dendritic spine morphogenesis by modulating actin cytoskeleton organization. The formation and plasticity of the dendritic spines are essential for normal brain function. Among the alternatively spliced βPix isoforms, βPix-b and βPix-d are expressed specifically in neurons. Our previous studies using cultured hippocampal neurons identified the roles of βPix-b and βPix-d in spine formation and neurite development, respectively. Here, we analyzed the *in vivo* role of the neuronal βPix isoforms in brain development and function by using βPix neuronal isoform knockout (βPix-NIKO) mice, in which the expression of the βPix-b and βPix-d isoforms is blocked, while the expression of the ubiquitous βPix-a isoform is maintained. Loss of the neuronal βPix isoforms leads to reduced activity of Rac1 and Cdc42, decreased dendritic complexity and spine density, and increased GluN2B and Ca^2+^/calmodulin-dependent protein kinase IIα expression in the hippocampus. The defects in neurite development, dendritic spine maturation, and synaptic density in cultured βPix-NIKO hippocampal neurons were rescued by the expression of βPix-b or βPix-d. In behavioral studies, βPix-NIKO mice exhibited robust deficits in novel object recognition and decreased anxiety levels. Our findings suggest that neuronal morphogenetic signaling by the neuronal βPix isoforms contributes to normal behaviors.

## Introduction

Dendritic spines are actin-rich, protrusive structures that primarily mediate excitatory synaptic transmission (Matus et al. 1982). Immature dendritic protrusions, known as filopodia, appear on the dendritic arbor and mature into spines with well-defined head and neck structures (Harris 1999; Hering and Sheng 2001; Newey et al. 2005). Morphogenesis of dendritic spines is crucial for synapse formation and plasticity, which underlie normal cognitive functions such as learning and memory (Nimchinsky et al. 2002; Alvarez and Sabatini 2007). Impairments in spine and synapse development are associated with numerous neurological disorders, including autism spectrum disorder (ASD), attention-deficit hyperactivity disorder (ADHD), and schizophrenia (Fiala et al. 2002; Penzes et al. 2011).

Neuronal morphogenesis, including neurite extension, dendritic spine formation, and synaptogenesis, typically involves the reorganization of the actin cytoskeleton (Fischer et al. 1998; Luo 2002). Members of the Rho family of small GTPases, including Rac, Cdc42, and Rho, play distinct roles in regulating the actin cytoskeleton and are involved in actin polymerization and actomyosin contractility (Etienne-Manneville and Hall 2002). Rho GTPases function as molecular switches, cycling between active GTP-bound forms and inactive GDP-bound forms. Guanine nucleotide exchange factors (GEFs) activate Rho GTPases by catalyzing the exchange of GDP for GTP (Schmidt and Hall 2002). Some GEFs have been reported to modulate neuronal morphology, synaptic plasticity, and cognitive function through their GTPase-regulating activity (Kiraly et al. 2010; Miller et al. 2013; De Filippis et al. 2014).

**Figure 1.**
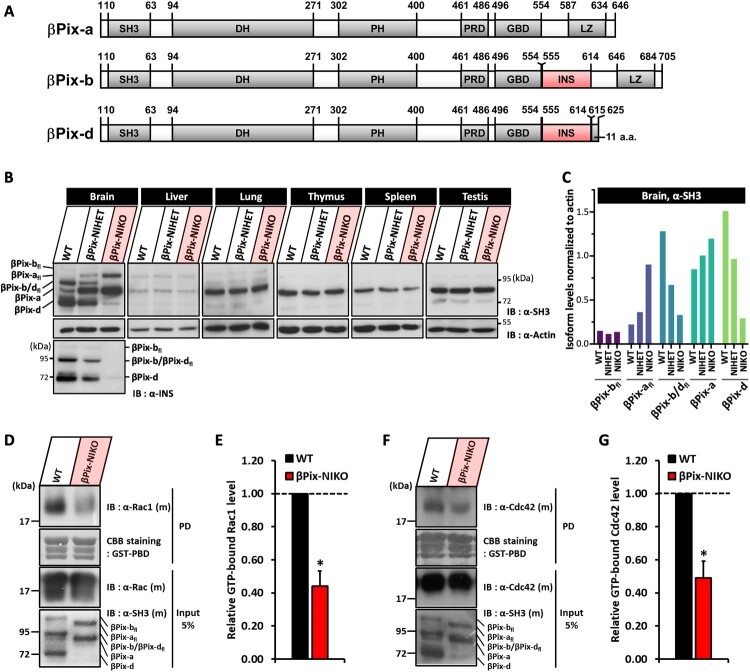
Absence of neuronal βPix isoforms leads to a significant reduction in active Rac1 and Cdc42 in the hippocampus. A. Domain structure of βPix-a, -b, and -d isoforms. SH3 = Src homology 3 domain, DH = Dbl homology domain, PH = Pleckstrin homology domain, PRD = Proline-rich domain, GBD = GIT1-binding domain, LZ = Leucine zipper domain, INS = novel insert region, 11 a.a. = additional 11 amino acids region. B. Expression patterns of βPix-a, -b, and -d in 4-week-old wild type (WT), βPix neuronal isoform heterozygous (βPix-NIHET), and βPix neuronal isoform knockout (βPix-NIKO) mice. βPix-a_fl_, βPix-b_fl_, and βPix-d_fl_ indicate the full-length form of each isoform, which contain an additional N-terminal region. Equal amounts of protein (15 μg) were loaded into each lane and probed with anti-SH3 and anti-INS antibodies. C. Quantitation of individual βPix isoform levels in the brain tissue, shown in (B). The isoform levels detected by the anti-SH3 antibody were normalized to actin. D. Representative blots of GTP-bound Rac1 isolated by the PBD pull-down assay in the hippocampus of 5-week-old mice. E. Quantification of (D). βPix-NIKO mice show reduced ratio of GTP-Rac1 to total Rac1. F. Representative blots of GTP-bound Cdc42 isolated by the PBD pull-down assay in the hippocampus of 5-week-old mice. G. Quantification of (F), showing the ratio of GTP-Cdc42 to total Cdc42. Data in (E) and (G) are from 3–4 independent experiments. *P < 0.05.

β-PAK-interacting exchange factor (βPix) acts as a Rho GEF that specifically activates Rac1 and Cdc42 (Bagrodia et al. 1998; Koh et al. 2001). It is an important regulator of cell migration and direction sensing in the immune system (Volinsky et al. 2006; Eitel et al. 2008) and cell adhesion, spreading, and cell–cell contact formation in cancer cells (Ahn et al. 2003; Flanders et al. 2003; Stevens et al. 2014). βPix has also been reported to be a regulator of spine morphogenesis, synapse formation, and neurite development in neurons (Park et al. 2003; Zhang et al. 2005; Saneyoshi et al. 2008; Kwon et al. 2019). We previously identified alternatively spliced isoforms of βPix: βPix-a, βPix-b, and βPix-d (Figure 1(A)). While βPix-a is ubiquitously expressed in most tissues, βPix-b and βPix-d are primarily expressed in the brain, specifically in neurons (Oh et al. 1997; Kim et al. 2000; Rhee et al. 2004). Although most studies on the neuronal role of βPix have focused on the ubiquitous βPix-a isoform, our recent studies have revealed the specific roles of the neuronal isoforms, βPix-b and βPix-d, in neurite formation and dendritic spine development in cultured hippocampal neurons (Shin et al. 2019; Kwon et al. 2020) and peripheral axon regeneration in sciatic nerves (Jeon et al. 2023). The neuronal βPix isoforms contain a distinct region called the insert (INS), which is not present in βPix-a. INS regulates spine morphogenesis through Src-dependent phosphorylation (Shin et al. 2019).

To investigate the roles of the neuronal βPix isoforms in brain development and behavior, we used a mouse model lacking expression of the neuronal βPix-b and βPix-d isoforms. In the βPix neuronal isoform knockout (βPix-NIKO) mice, we observed decreased activity of Rac1 and Cdc42 in the hippocampus, along with simplified dendritic arbors and reduced dendritic spine density in hippocampal pyramidal neurons *in vivo*. Consistent with these morphological phenotypes, βPix-NIKO mice exhibited defects in novel object recognition and lower anxiety levels. These data suggest that the neuronal βPix isoforms are necessary for the normal development of neuronal structures in the hippocampus and regulate behaviors associated with neurological diseases.

## Materials and methods

### βPix-NIKO mice

The generation of βPix-NIKO mice has been previously described (Kwon et al. 2020). Mice were bred and housed in facilities at Seoul National University and Dong-A University, adhering to approved protocols (SNU-130225-11-3, DIACUC-22-30). Euthanasia was performed via CO_2_ inhalation in adult mice and decapitation in neonates. Homozygous βPix-NIKO mice were produced by interbreeding heterozygous mice, with genotypes confirmed by PCR. The PCR primers used were: forward primer 5′-AGCACAGTTGACGTTGCTTTCTGTC-3′, WT-specific reverse primer 5′-AAAGCCCATCAGGTACTCACTGGAC-3′, and Δ-specific reverse primer 5′-AAACTATCAGTCTGCCCTCACCCAC-3′. The annealing temperature was 58°C, and PCR products were amplified over 40 cycles.

### Primary antibodies

Polyclonal rabbit antibodies were generated against the βPix SH3 and INS domains as described previously (Oh et al. 1997; Kim et al. 2000). The following monoclonal mouse antibodies were used: actin (clone AC-40, Sigma), Ca^2+^/calmodulin-dependent protein kinase (CaMK) IIα (clone CBα-2, Zymed Laboratories), Cdc42 (clone 44/CDC42, BD Transduction Laboratories^TM^), Gephyrin (clone mAb7a, Synaptic Systems), GluA1 (clone N355/1, Abcam), GluA2 (clone 6C4, Zymed Laboratories), PSD-95 (clone 7E3-1B8, Thermo Scientific), Rac1 (clone 23A8, Millipore), synaptophysin (clone SY38, Chemicon), α-tubulin (clone DM1A, Abcam), and β3-tubulin (clone TU-20, Chemicon). Polyclonal rabbit antibody against GluN2B (Chemicon), polyclonal guinea pig antibody against VGAT (Synaptic Systems), and polyclonal rabbit antibody against VGLUT1 (Synaptic Systems) were also commercially purchased.

### Western blot analysis

Tissues from 4-, 5-, and 12-week-old mice were homogenized in ice-cold buffer, and lysates were obtained after centrifugation. Protein concentrations were determined using the Bradford assay. Equal amounts of protein were subjected to SDS-PAGE, transferred to PVDF membranes, and blocked with 3% BSA. Primary antibodies were applied for 1 h at room temperature, followed by the addition of secondary antibodies. Blots were analyzed via enhanced chemiluminescence, with α-tubulin and actin serving as the loading controls.

### Nissl staining

Thirteen-week-old mice were anesthetized and perfused with 3.7% PFA. The brains were cryoprotected, embedded in O.C.T. compound (Tissue-Tek), and sectioned at 25 μm. Sections were stained with cresyl violet, dehydrated, cleared in xylene, and coverslipped using Permount^TM^ medium (Fisher Scientific). Images were captured with a Zeiss Lumar V12 microscope using a 0.8× (NeoLumar S) objective lens.

### *In vivo* glutathione S-transferase (GST) pull-down assay

Brain and hippocampus homogenates from 5- and 8-week-old mice were prepared in ice-cold homogenization buffer (50 mM HEPES, pH 7.4, 150 mM NaCl, and protease and phosphatase inhibitors) and centrifuged. Lysates were incubated with GST-Rac/Cdc42 (p21) binding domain proteins that were pre-bound to Glutathione Sepharose 4B (Amersham Pharmacia). After incubation and washing, samples were analyzed by SDS-PAGE and immunoblotting.

### Golgi staining

Five- and 12-week-old mice were perfused, and brains were stained using the FD Rapid GolgiStain kit (NeuroTechnologies). Subsequently, 150-μm thick sections were obtained using a vibratome, mounted, and imaged with a Zeiss Axiovert 200M microscope equipped with a Zeiss Axiocam HRm CCD camera using a 40×, 0.6 NA LD Achroplan objective and a 100×, 1.40 NA Plan-Apochromat objective. ImageJ software was used for quantification, which was performed in a blinded manner.

### Sholl analysis and spine density assay

Pyramidal neurons from the CA1 hippocampal regions were analyzed. Sholl analysis was performed using a transparent grid with 10 μm concentric rings to measure the number of ring intersections. Spine density was assessed by counting spines along randomly selected dendritic segments. Protrusions were categorized as filopodia or spines with distinguishable heads (Kim et al. 2024).

### Primary mouse hippocampal neuron culture and transfection

Mouse hippocampal neurons were prepared from P0–1 mouse pups as previously described (Beaudoin et al. 2012). Hippocampal tissues were treated with papain and DNase, followed by mechanical dissociation. Neurons were plated in MEM supplemented with glucose, sodium pyruvate, antibiotics, L-glutamine, and 10% fetal bovine serum. After 4 h, the medium was replaced with a growth medium. Neurons were maintained for up to 21 days in a 5% CO_2_ incubator, with the medium replaced every 4–7 days. Transient transfection was performed at 7 days *in vitro* (DIV) using the calcium phosphate method (CalPhos Transfection Kit, Calbiochem). GFP-βPix-b and GFP-βPix-d constructs were generated by subcloning coding regions into pEGFP-N1 (Clontech) using PCR.

### Immunocytochemistry and analysis of the spine and synapse morphology

Immunocytochemistry was performed as described previously (Kwon et al. 2020; Kim et al. 2023). Imaging was performed using a Zeiss Axiovert 200M, as described above, or Zeiss LSM700 equipped with 40×, 1.20 NA C-Apochromat (Carl Zeiss), maintaining constant settings. Multiple images were collaged for identifying neurons with large arborization spanning more than one field of view. ImageJ software was used for dendrite analysis and spine assays, with protrusions categorized as filopodia or dendritic spines. Synaptic protein puncta were analyzed by measuring the fluorescence intensity.

### Behavioral tests

For the novel object recognition assay, mice explored two identical objects in an open-field for 10 min. During the test phase, one object was replaced, and exploration time was measured. Object exploration was defined as instances where a mouse’s nose was within 2 cm of the object. For the elevated plus-maze, mice were placed in an elevated plus-maze with two open arms and two closed arms. Mice were allowed to explore the maze for 8 min, and the time spent in each arm was recorded to assess anxiety-like behavior.

### Statistics

All data are expressed as the mean±standard error of the mean. All analyses involved a minimum of three independent experiments, and data were evaluated using Student’s *t*-test.

## Results

### Neuronal βPix isoform knockout mice display loss of βPix-b and βPix-d expression in the brain

Alternatively spliced isoforms of βPix (Figure 1(A)) exhibit distinct expression patterns in different organs (Shin et al. 2019). Western blot analysis using antibodies against the SH3 and INS domains confirmed that βPix-b and βPix-d expression is specific to the brain tissue (wild type [WT] in Figure 1(B)). In the brain, βPix-b and βPix-d were more abundant than the ubiquitous βPix-a isoform (Figure 1(B)). To investigate neuronal βPix isoform-specific function, we used βPix-NIKO mice that we had generated previously (Kwon et al. 2020). Exon 19, encoding the neuronal isoform-specific INS region, was deleted in βPix-NIKO, eliminating the expression of the βPix-b and βPix-d isoforms (Supplementary Fig. 1A). Western blot using the INS antibody showed a complete loss of βPix-b and βPix-d in whole brain extracts from the βPix-NIKO mice (Figure 1(B)). Deletion of the INS region in βPix-b, as detected by the SH3 antibody, resulted in increased expression of βPix-a (Figure 1(C)). These βPix expression patterns were consistent across different regions of the central nervous system and in cultured hippocampal neurons, independent of the neuronal developmental stage (Supplementary Fig. 1B and 1C). βPix-a expression levels in other organs were maintained in βPix-NIKO mice (Figure 1(B)). The βPix-NIKO mice were viable and fertile, and body weight, brain weight, brain size, and brain histology did not significantly differ between WT and βPix-NIKO mice (Supplementary Fig. 2). These findings suggest that this mouse model is suitable for studying the neuronal functions of βPix-b and βPix-d.

### Active Rac1 and Cdc42 are reduced in the hippocampus of βPix-NIKO mice

βPix shows GEF activity toward Rac1 and Cdc42, and we have previously reported that βPix-b has a higher GEF activity than βPix-a in cultured neurons (Shin et al. 2019). Src-dependent phosphorylation in the INS region, which is absent in βPix-a, is responsible for the enhanced GEF activity of βPix-b. Hence, we examined whether the loss of the neuronal βPix isoforms affects the levels of GTP-bound Rac1 and Cdc42 in the hippocampus. The hippocampi of βPix-NIKO mice showed a 56% decrease in Rac1-GTP levels (Figure 1(D,E)) and 51% decrease in Cdc42-GTP levels (Figure 1(F,G)), indicating that the loss of neuronal βPix isoforms leads to reduced Rac1 and Cdc42 signaling in the hippocampus.

### Dendritic complexity and spine density are decreased in the hippocampal pyramidal neurons of βPix-NIKO mice

**Figure 2.**
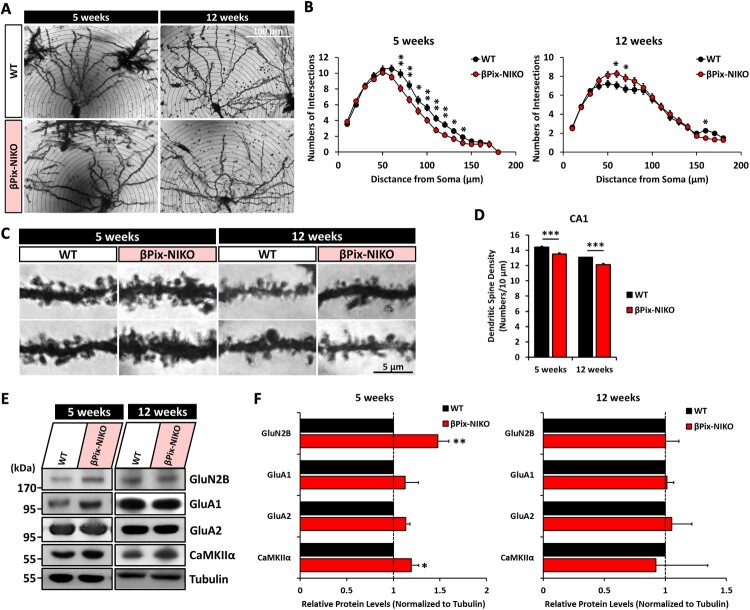
βPix neuronal isoform KO (βPix-NIKO) mice display defective neuronal morphology and increased synaptic protein expression in hippocampal tissues. A. Representative images of basal dendrites of pyramidal neurons in hippocampal CA1 regions of 5- and 12-week-old WT and βPix-NIKO littermate mice. B. Sholl analysis of (A) shows reduced neuronal complexity in 5-week-old βPix-NIKO mice than in wild type (WT) controls. C. Representative images of dendritic spines of pyramidal neurons in the hippocampal CA1 regions. D. Quantification of (C). βPix-NIKO mice show a significantly lower spine density than WT mice. E. Representative blots of synaptic proteins. F. Quantification of (E) showing the relative levels of synaptic proteins normalized to tubulin expression. Protein levels of GluN2B and CaMKIIα were increased in the hippocampus of 5-week-old βPix-NIKO mice. *n* = 55–57 neurons per group for (A) and (B). *n* = 108–183 dendritic segments per group for (C) and (D). *P < 0.05, **P < 0.01, and ***P < 0.001.

Dendritic complexity is regulated by Rac1 and Cdc42 GTPases and correlates with the number and distribution of synaptic inputs that a neuron receives, contributing to synaptic plasticity and computation (Jan and Jan 2001; Poirazi and Mel 2001). Because the activities of Rac1 and Cdc42 were reduced in βPix-NIKO mouse brains, we examined whether dendritic arborization was affected in βPix-NIKO mice. Dendritic growth and branching were determined using Sholl analysis, which quantifies the number of dendrite intersections by drawing concentric circles of increasing diameter around the cell body. On analyzing the basal dendrites of CA1 pyramidal neurons in the Golgi-stained hippocampus, dendritic trees in 5-week-old juvenile βPix-NIKO animals were found to be simplified than those in age-matched WT mice, while 12-week-old adult WT and βPix-NIKO mice did not demonstrate marked differences in dendritic arborization (Figure 2(A,B)).

Next, we investigated dendritic spine formation in βPix-NIKO mice. The involvement of βPix-b in dendritic spine formation has been demonstrated in cultured hippocampal neurons (Shin et al. 2019). To examine the role of the neuronal βPix isoforms *in vivo*, we measured the dendritic spine density in Golgi-stained hippocampal CA1 pyramidal neurons from WT and βPix-NIKO mice. We found a slight but significant decrease in the dendritic spine density in 5- and 12-week-old βPix-NIKO mice (Figure 2(C,D)). These data indicate that the neuronal βPix isoforms are required for the normal development of hippocampal pyramidal neurons *in vivo*.

### GluN2B and CaMKIIα expression is increased in the hippocampus of juvenile βPix-NIKO mice

Given the decreased spine density in hippocampal CA1 pyramidal neurons of βPix-NIKO mice, we examined whether the proteins involved in excitatory neurotransmission were affected. We assessed hippocampal homogenates from WT and βPix-NIKO mice using western blot analysis. GluN2B, a subunit of NMDA-type glutamate receptors (NMDARs), showed significantly increased expression in the hippocampus of 5-week-old βPix-NIKO mice than in WT mice, but not in 12-week-old βPix-NIKO mice (Figure 2(E,F)). Furthermore, we observed increased expression of CaMKIIα, a kinase activated by Ca^2+^ influx through NMDARs, in the hippocampus of 5-week-old βPix-NIKO mice than in WT controls. However, this increase was not evident in the hippocampus of 12-week-old βPix-NIKO mice (Figure 2(E,F)). We did not observe consistent changes in the levels of GluA1 and GluA2, which are subunits of the AMPA-type glutamate receptors, in either 5- or 12-week-old βPix-NIKO mice (Figure 2(E,F)). Notably, βPix-b and βPix-d were detected in the synaptosomes and synaptic membrane fractions from 5-week-old mice. GluN2B was enriched in the postsynaptic density (PSD) fractions from the hippocampus of βPix-NIKO mice compared to those from WT controls (Supplementary Fig. 3). Therefore, deficiency of neuronal βPix isoforms leads to morphological and molecular defects in dendritic spines, suggesting that these defects may impair synaptic function in the hippocampus during adolescent development.

### βPix-b and βPix-d are required for normal neurite structure in cultured neurons

**Figure 3.**
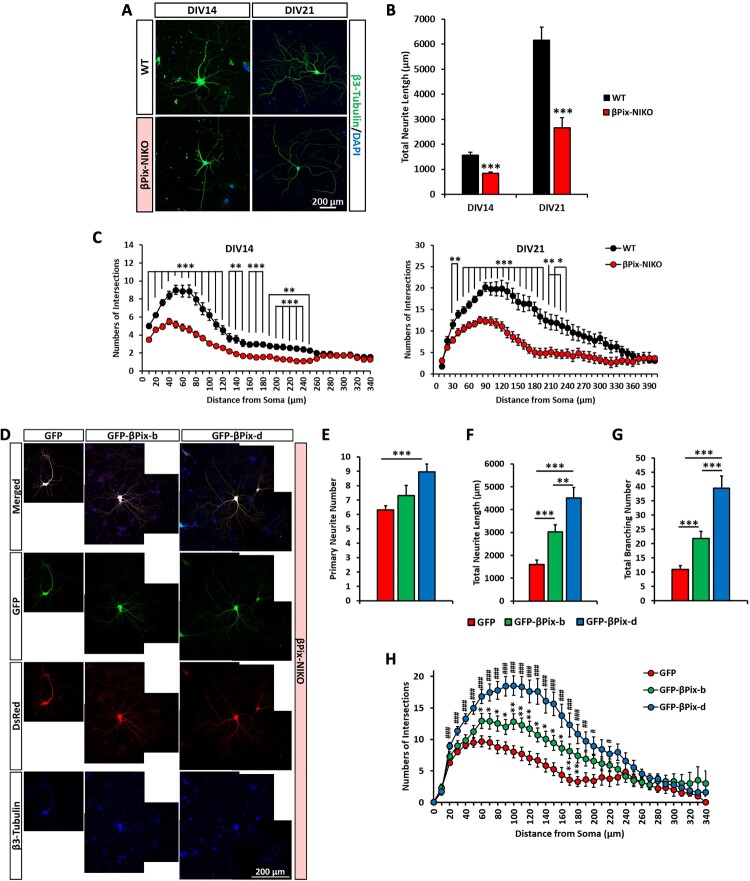
βPix-b and βPix-d are required for normal neurite morphogenesis. A. Representative images of wild type (WT) and βPix neuronal isoform KO (βPix-NIKO) hippocampal neuron cultures fixed at days *in vitro* (DIV)14 and DIV21. B. Quantification of (A). Total neurite length is significantly shorter in βPix-NIKO neurons than in WT neurons. C. Sholl analysis of (A) shows simplified dendritic arborizations in βPix-NIKO neurons than those in WT neurons. D. Representative images of βPix-NIKO hippocampal neurons co-transfected with GFP, GFP-βPix-b, or GFP-βPix-d and DsRed at DIV7 and analyzed by double immunofluorescence with antibodies against GFP and β3-tubulin at DIV14. E–G. Primary neurite numbers (E) are significantly increased in βPix-NIKO neurons expressing βPix-d. Total neurite length (F) and branching numbers (G) are significantly increased in βPix-NIKO neurons expressing βPix-b or βPix-d. H. Sholl analysis of (D) demonstrates more complex dendritic arborizations in βPix-NIKO neurons expressing βPix-b or βPix-d than those in GFP-expressing βPix-NIKO neurons. *n* = 62–66 neurons at DIV14 and *n* = 50–51 neurons at DIV21 for (A)–(C). *P < 0.05, **P < 0.01, and ***P < 0.001. *n* = 45–60 neurons per group for (D)–(H). In (H), *P < 0.05, **P < 0.01, ***P < 0.001 (GFP vs. GFP-βPix-b), #*P* < 0.05, ##*P* < 0.01, ###*P* < 0.001 (GFP vs. GFP-βPix-d).

To directly determine whether each βPix neuronal isoform is required for dendritic arborization and spine development, we used cultured hippocampal neurons to assess the ability of the individual neuronal isoforms in rescuing the βPix-NIKO phenotypes. We previously reported that βPix-NIKO neuronal cultures display reduced early stage neurite formation at DIV4 (Kwon et al. 2020). In this study, neurons were examined in later stages, at DIV14 and DIV21, to assess dendritic complexity and synapse formation in relation to the βPix-NIKO phenotypes observed *in vivo* (Figure 2(A–D)). Immunostaining for β3-tubulin revealed that loss of neuronal βPix isoforms led to reduced total neurite length and neuronal complexity at DIV14 and DIV21 (Figure 3(A–C)), consistent with the *in vivo* results (Figure 2(A,B)).

To assess the involvement of individual neuronal βPix isoforms in dendritic arborization, hippocampal neurons cultured from βPix-NIKO mice were transfected with GFP, GFP-βPix-b, or GFP-βPix-d, alongside DsRed to label transfected cells, at DIV7. Immunostaining for β3-tubulin at DIV14 showed that βPix-NIKO neurons expressing GFP-βPix-d exhibited a marked increase in primary neurite numbers compared to control-transfected βPix-NIKO neurons (GFP) (Figure 3(D–E)). Total neurite length, branching number, and neuronal complexity were also significantly increased in βPix-NIKO neurons transfected with βPix-b or βPix-d (Figure 3(F–H); βPix expression levels shown in Supplementary Figure 4). Notably, the phenotypic rescue by βPix-d was significantly stronger than that by βPix-b, suggesting that different neuronal βPix isoforms may vary in their ability to promote neurite morphogenesis.

### βPix-b and βPix-d promote dendritic spine development

**Figure 4.**
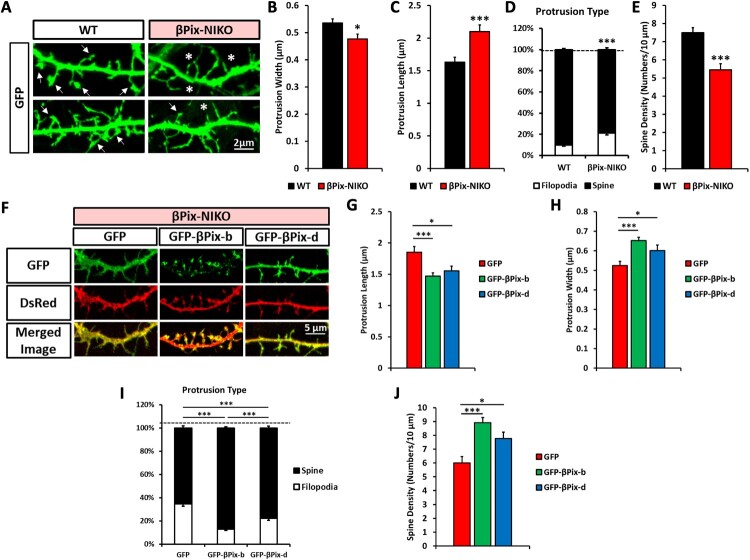
βPix-b and βPix-d are essential for dendritic spine morphology and density. A. Representative images of wild type (WT) and βPix neuronal isoform KO (βPix-NIKO) cultured hippocampal neurons transfected with GFP at days *in vitro* (DIV)7 and fixed at DIV14. Arrows indicate dendritic protrusions with heads, and asterisks indicate those without heads. Quantification in (B)–(E). B–E. The protrusions were significantly longer (C) in βPix-NIKO neurons than in WT neurons. Conversely, the width of protrusions (B), percentage of dendritic spines (D), and dendritic spine density (E) were significantly reduced in βPix-NIKO neurons. Filopodia were identified as long and thin structures with a length of 3 μm or more, width of 0.3 μm or less, and lacking heads. The remaining protrusions were classified as spines. F. Representative images of βPix-NIKO hippocampal neurons co-transfected with GFP, GFP-βPix-b, or GFP-βPix-d and DsRed at DIV7 and analyzed at DIV14. Quantification in (G)–(J). G–H. Dendritic protrusions in βPix-NIKO neurons expressing βPix-b or βPix-d were significantly shorter and thicker than in those expressing GFP. I. The percentage of filopodia was significantly reduced by expression of βPix-b or βPix-d in βPix-NIKO neurons. J. Dendritic spine density was significantly increased in βPix-NIKO neurons expressing βPix-b or βPix-d than that in those expressing GFP. *n* = 683–708 protrusions per group for (A)–(E) and *n* = 600–627 protrusions per group for (F)–(J). *P < 0.05, **P < 0.01, and ***P < 0.001.

To further examine the role of the neuronal βPix isoforms in dendritic spine development, hippocampal neurons cultured from P0–1 WT and βPix-NIKO littermates were transfected with GFP at DIV7 to visualize the cellular morphology and analyzed at DIV14 for dendritic spine morphology (Figure 4(A)). We found a significant increase in the length and decrease in the width of dendritic protrusions in βPix-NIKO neurons compared to those in WT neurons (Figure 4(B,C)), consistent with a higher proportion of immature filopodia among dendritic protrusions in βPix-NIKO neurons (Figure 4(D)). Conversely, we detected a significant decrease in dendritic spine density in βPix-NIKO neurons (Figure 4(E)), similar to that observed in the Golgi-stained hippocampal tissue (Figure 2).

We have previously reported that βPix-b contributes to dendritic spine development (Shin et al. 2019). Hence, we investigated whether defective spine formation in the βPix-NIKO neurons could be reversed by expressing βPix-b and βPix-d. Expression of either GFP-βPix-b or GFP-βPix-d significantly decreased the protrusion length and increased the protrusion width at DIV14 compared to expression of GFP only in the control (Figure 4(F–H)). Each neuronal βPix isoform significantly reduced the percentage of filopodia and increased the dendritic spine density (Figure 4(I,J)), indicating that the neuronal βPix isoforms influence the maturation of dendritic spines. Notably, βPix-b rescued the defects in spine maturation and density in the βPix-NIKO neurons more effectively than βPix-d, suggesting that βPix-b may be the primary βPix isoform regulating dendritic spine formation. Importantly, these experiments demonstrate that the neuronal βPix isoforms are required for dendritic arborization and spine development.

### βPix-b and βPix-d promote synaptic development

**Figure 5.**
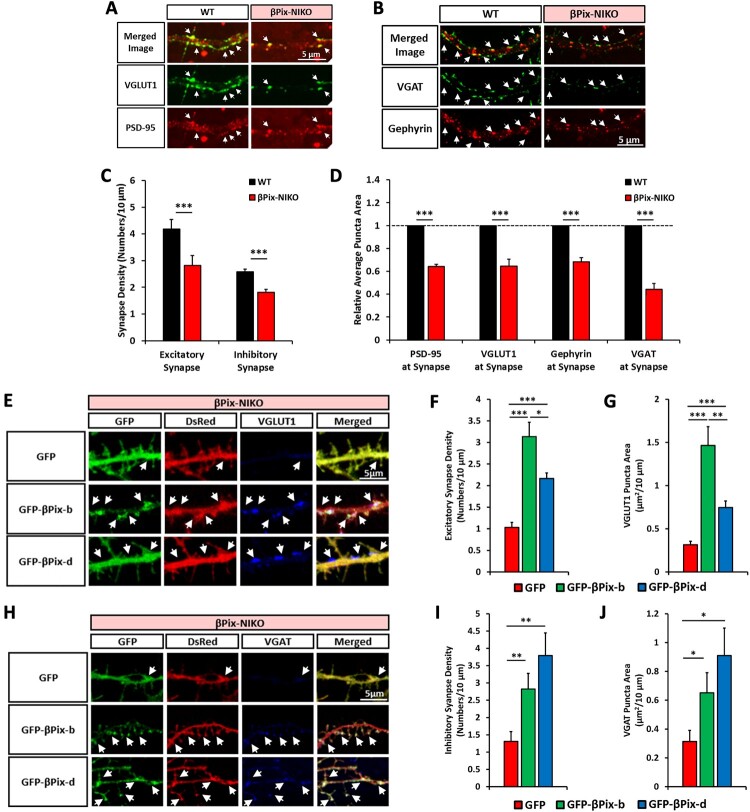
βPix-b and βPix-d are required for excitatory and inhibitory synapse development. A. Representative images of hippocampal neurons stained for VGLUT1, an excitatory presynaptic marker, and PSD95, an excitatory postsynaptic marker at days *in vitro* (DIV)14. Arrowheads denote VGLUT1-positive PSD-95 puncta, indicating excitatory synapses. Quantification in (C) and (D). B. Representative images of hippocampal neurons stained for VGAT, an inhibitory presynaptic marker, and Gephyrin, an inhibitory postsynaptic marker at DIV14. Arrowheads denote VGAT-positive Gephyrin puncta, indicating inhibitory synapses. Quantification in (C) and (D). C. Densities of VGLUT1-positive PSD-95 puncta and VGAT-positive Gephyrin puncta were decreased in βPix neuronal isoform KO (βPix-NIKO) neurons than in wild type (WT) neurons. D. PSD-95, VGLUT1, Gephyrin, and VGAT puncta area at synapse were reduced in βPix-NIKO neurons than in WT neurons. E. Representative images of βPix-NIKO hippocampal neurons co-transfected with GFP, GFP-βPix-b, or GFP-βPix-d and DsRed at DIV7 and analyzed by double immunofluorescence with antibodies against GFP and VGLUT1 at DIV14. Quantification in (F) and (G). F–G. Excitatory synapse density (F) and VGLUT1 puncta area (G) were increased by the expression of βPix-b or βPix-d in βPix-NIKO neurons. H. Representative images of hippocampal neurons co-transfected with GFP, GFP-βPix-b, or GFP-βPix-d and DsRed at DIV7 and analyzed by double immunofluorescence with antibodies against GFP and VGAT at DIV14. Quantification in (I) and (J). I–J. Inhibitory synapse density (I) and VGAT puncta area (J) were increased by the expression of βPix-b or βPix-d in βPix-NIKO neurons. *n* = 62–80 neurons for (A)–(D), *n* = 61–62 neurons for (E)–(G) and *n* = 24 neurons for (H)–(J). *P < 0.05, **P < 0.01, and ***P < 0.001.

To determine whether abnormal dendritic spine development caused by loss of βPix-b and βPix-d affects synapse development, we examined the excitatory and inhibitory synapse densities (Figure 5). DIV14 hippocampal neurons cultured from P0–1 WT and βPix-NIKO littermate mice were stained for PSD-95 (postsynaptic marker) and VGLUT1 (presynaptic marker) to visualize excitatory synapses (Figure 5(A)) and Gephyrin (postsynaptic marker) and VGAT (presynaptic marker) to visualize inhibitory synapses (Figure 5(B)). Synapses were defined as immunofluorescent puncta that were positive for both presynaptic and postsynaptic markers. The βPix-NIKO neurons displayed a significant reduction in both excitatory and inhibitory synaptic densities (Figure 5(C)). The puncta sizes, when immunostained with individual synaptic markers, PSD-95, VGLUT1, Gephyrin, and VGAT, were also markedly decreased in the βPix-NIKO neurons (Figure 5(D)).

We then investigated whether the neuronal βPix isoforms promote synapse formation and maturation by expressing each isoform in βPix-NIKO hippocampal neurons. Excitatory and inhibitory synaptic densities were determined by quantifying the number of VGLUT1 (Figure 5(E)) and VGAT (Figure 5(H)) puncta, respectively, in the DsRed-positive transfected neurons. We found that expressing either βPix-b or βPix-d significantly increased both the excitatory and inhibitory synapse densities (Figure 5(F,I)), consistent with previous observations that βPix is involved in excitatory (Zhang et al. 2005) and inhibitory synapse formation (Smith et al. 2014). Moreover, VGLUT1 and VGAT puncta areas per 10 μm neurite were significantly increased by the expression of GFP-βPix-b or GFP-βPix-d in βPix-NIKO neurons than those in the GFP control (Figure 5(G,J)). Importantly, βPix-b expression reversed the deficits in excitatory synapse density in βPix-NIKO neurons more effectively than βPix-d expression (Figure 5(F)), indicating that the βPix-b isoform plays a major role in dendritic spine and excitatory synapse maturation. In contrast, expression of βPix-d was more effective in reversing the deficits in inhibitory synapse density than expression of βPix-b (Figure 5(I)). Analyses for excitatory and inhibitory synapses were conducted using the same transfection method; therefore, the contrasting phenotypes are likely due to the differential molecular functions of the two neuronal isoforms. Investigation of the subcellular localization of exogenous βPix-b or βPix-d in the βPix-NIKO neurons showed that GFP-βPix-b was mainly present in the head of the dendritic spine, whereas GFP-βPix-d was present in the dendritic shaft and spine neck (Figure 5(E,H)). This is consistent with the findings of our previous study that βPix-b preferentially localizes to the dendritic spines than βPix-a in cultured rat hippocampal neurons, and that this localization requires the leucine zipper domain, which is absent in βPix-d (Shin et al. 2019). Thus, βPix-b and βPix-d are differentially localized in the neurons and regulate the formation of different types of synapses in an isoform-specific manner.

### βPix-NIKO mice show impaired recognition memory and low anxiety levels

**Figure 6.**
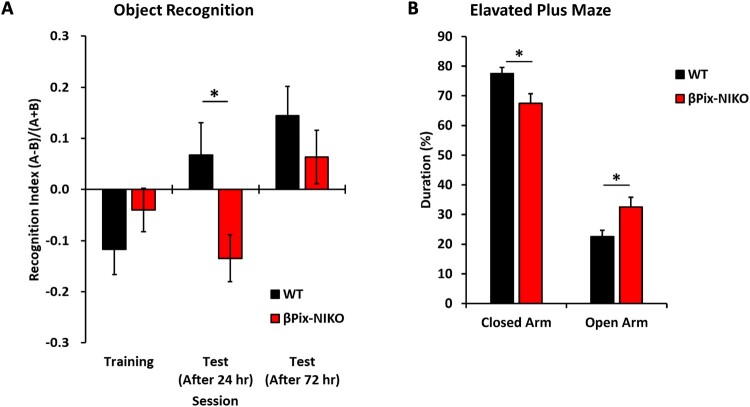
Neuronal βPix isoform KO (βPix-NIKO) mice show impaired recognition memory and low anxiety levels. A. βPix-NIKO mice spent less time exploring a novel object than wild type (WT) mice. In the recognition index, A represents the amount of time spent interacting with a novel object and B with a familiar object. During training, both genotypes showed a preference for the familiar object side (B), likely due to contextual factors such as object placement. B. βPix-NIKO mice spent less time in closed arms and more time in open arms in the elevated plus maze test than WT mice. *n* = 12 10–12-week-old mice for WT and *n* = 10 10–12-week-old mice for βPix-NIKO. *P < 0.05.

Dendritic spine morphogenesis and synapse formation are required for normal cognitive function, and cognitive deficits are associated with neurological disorders in humans, such as schizophrenia, ADHD, and Alzheimer’s disease (Barch 2005; Germano and Kinsella 2005; Doyle 2006). Since our molecular and cellular studies of βPix-NIKO mice indicated hippocampal synaptic defects, we examined whether the deficiency of neuronal βPix isoforms affects cognitive functions. In βPix-NIKO mice, spatial learning assessed by the Morris water maze test was generally intact for learning the localization of a hidden platform (Supplementary Figure 5A). Total distances traveled during the open-field test indicated that motor abilities of the βPix-NIKO mice were normal for open-field locomotion (Supplementary Fig. 5B). In contrast, the βPix-NIKO mice showed impaired performance in the novel object recognition test in the test session conducted 24 h after training (Figure 6(A)). WT mice spent more time exploring the novel object than the familiar object, whereas βPix-NIKO mice demonstrated no preference for the novel object. No significant genotype differences were found in the test conducted 72 h after training, indicating that βPix-NIKO mice are slower in recognizing a novel object than WT mice. Novel object recognition is a form of declarative memory that is dependent on intact CA1 hippocampal function (Rampon and Tsien 2000; Hammond et al. 2004). These data suggest that the molecular and morphological defects in the hippocampal neurons of βPix-NIKO mice could contribute to the deficits in recognition memory for novelty.

Next, we determined whether the βPix-NIKO mice showed deficits in different behavioral phenotypes mainly associated with brain regions other than the hippocampus. First, baseline anxiety-like behavior was assessed in WT and βPix-NIKO mice using an elevated plus maze test. βPix-NIKO mice spent significantly longer time in the open arms of the elevated plus maze than WT mice (Figure 6(B)), indicating that βPix-NIKO mice have low anxiety levels. These data are consistent with our results that the βPix-NIKO mice showed significantly lower levels of freezing than WT mice during conditioning in the trace fear conditioning test (Supplementary Fig. 6A), whereas freezing levels during testing were not altered (Supplementary Fig. 6B). These findings suggest that baseline anxiety levels in βPix-NIKO mice are lower than those in WT mice, although they appear to form normal trace fear conditioning memory. Therefore, the loss of neuronal βPix isoforms causes behavioral impairments consistent with defective neuronal morphogenesis, indicating that the neuronal βPix isoforms are vital for neural circuit formation and function.

## Discussion

βPix-b and βPix-d are specifically expressed in neurons, and their neuronal expression levels exceed those of the ubiquitously expressed βPix-a. Although previous studies have examined βPix functions in neuronal culture models, their roles in brain development and function remain largely unexplored (Zhang et al. 2005; Shin et al. 2019; Kwon et al. 2020). We used a mouse model lacking neuronal βPix isoforms to characterize their roles in hippocampal neuronal morphology, connectivity, and specific behavioral phenotypes.

βPix-NIKO mice, in which βPix-b and βPix-d expression is eliminated, showed decreased levels of hippocampal neurite complexity and dendritic spine density, accompanied by lower levels of active Rac1 and Cdc42 in the hippocampus compared to WT mice. Because the Rho family of GTPases plays important roles in various aspects of neuronal development, the loss of neuronal βPix isoforms affects dendritic complexity and spine density, likely due to impaired Rac1 and Cdc42 signaling. In this study, we provide the first *in vivo* evidence that βPix-b and βPix-d play essential roles in neuronal development and activation of Rac1 and Cdc42 in the hippocampus.

In βPix-NIKO mice, βPix-a expression increases, likely because the deletion of exon 19 results in a protein identical to βPix-a instead of βPix-b. Hence, the observed phenotypes of the βPix-NIKO mice might be caused either by the loss of neuronal βPix isoforms or overexpression of βPix-a. Complementing βPix-b or βPix-d in the hippocampal neurons cultured from βPix-NIKO mice reversed the defects in neuronal morphogenesis and synapse development, confirming the necessity of the neuronal isoforms. Interestingly, expression of the individual neuronal βPix isoforms demonstrated isoform-specific functions, with each isoform selectively regulating different stages of neuronal development. These distinct molecular roles are consistent with previous findings that βPix-d regulates microtubule stability in developing neurites (Kwon et al. 2020; Jang et al. 2024), and that the role of βPix-b in spine morphogenesis is dependent on the leucine zipper domain, which is absent in βPix-d (Llano et al. 2015; Shin et al. 2019).

Ca^2+^-mediated activation of CaMKs, involved in NMDAR-dependent signaling, has been reported to promote phosphorylation of βPix at Ser516 in the Git1/βPix complex. This phosphorylation stimulates GEF activity to activate Rac1, which then promotes dendritic spine formation and stabilization (Saneyoshi et al. 2008). We found that both the neuronal βPix isoforms and GluN2B were enriched in the same PSD fraction, with increased expression levels of GluN2B and CaMKIIα in the βPix-NIKO hippocampi. This suggests that βPix has a specific role in NMDAR signaling, as previously indicated by Fiuza et al. (2013). However, increased GluN2B expression in βPix-NIKO mice, along with decreased spine formation, appear contradictory (Brigman et al. 2010) and may be due to presynaptic or postsynaptic effects resulting from the loss of βPix. βPix-mediated actin polymerization at synapses regulates vesicle localization in presynaptic terminals (Momboisse et al. 2009; Sun and Bamji 2011; Shirafuji et al. 2014); thus, the loss of the neuronal βPix isoforms potentially leads to homeostatic expression of GluN2B and CaMKIIα. Postsynaptically, decreased dendritic complexity and spine density in the βPix-NIKO hippocampi might induce compensatory expression of GluN2B.

Given the established role of dendritic spines in memory formation (Benarroch 2007), the learning deficits observed in the βPix-NIKO mice are not surprising. The mice showed intact spatial memory and learning for fear conditioning but displayed impaired novel object recognition. This aligns with the idea that different hippocampal synapses and pathways underlie various forms of memory (Ramos 2000; Antunes and Biala 2012).

Neurological disorders often involve cognitive disabilities, anxiety, and abnormal sensory sensitivity. Our findings indicate potential roles of βPix signaling in the pathogenesis of neurological disorders. Although no direct genetic link with neurological disorders has been identified for βPix, patients with deletions of chromosome bands 13q33-34, including *ARHGEF7*, exhibit mental retardation and microcephaly, suggesting that *ARHGEF7* is a candidate gene (Walczak-Sztulpa et al. 2008). Furthermore, βPix interacts with several risk factors for neurological disorders, including Shank (ASD and schizophrenia risk; Park et al. 2003; Zhou et al. 2016), Git (ADHD; Bagrodia et al. 1998; Won et al. 2011), and LRRK2 (Parkinson’s disease; Zimprich et al. 2004; Haebig et al. 2010). Studies investigating the association of other Rac/Cdc42 GEF genes, such as *ARHGEF6, KALRN, and TIAM1,* with neuropsychiatric disorders have further supported the involvement of βPix signaling in neuropsychiatric disease mechanisms (Mendoza-Naranjo et al. 2007; Youn et al. 2007; Ma et al. 2008; Kushima et al. 2012; Ramakers et al. 2012).

βPix, Kalirin-7, and Tiam1 are Rho-family GEFs that play roles in hippocampal synaptic transmission (Xie et al. 2011; Blanco-Suarez et al. 2014). βPix has unique features, such as forming a positive feedback loop with Pak and Git, suggesting tight spatial and temporal regulation of βPix-dependent signaling (Tolias et al. 2011). Future studies on circuit formation, behavior, and neurochemical mapping using Rho-family GEF mutant mouse lines may clarify the shared and distinct roles of GEFs in synaptic signaling pathways.

Our results provide neurobiological insights into how the loss of neuronal βPix isoforms leads to morphological and synaptic defects in the hippocampus and related behavioral abnormalities. Further investigation of neural circuits with complete absence of βPix-b and βPix-d may uncover the detailed molecular mechanisms of synapse development via these isoforms and how their dysfunction contributes to neuropsychiatric conditions.

## Supplementary Material

Supplemental Material
