# Supplementary material for: Loss of neuronal βPix isoforms impairs neuronal morphology in the hippocampus and causes behavioral defects
Source: Anim Cells Syst (Seoul). 2025 Jan 8;29(1):57–71. doi: 10.1080/19768354.2024.2448999 (PMC11722029; doi:10.1080/19768354.2024.2448999)
Supplement: Supplemental Material [file TACS_A_2448999_SM5991.zip › Supplementary Documents_R1.docx]

**Supplementary Document**

**Supplementary methods**

**Postsynaptic density (PSD) preparation from mouse brain**

Whole brain and hippocampal tissues were obtained from 5- and 12-week-old mice and experiments were performed as described previously ([Peca et al., 2011](#_3cqmetx)). For hippocampus samples, tissues from three mice were pooled to prepare each sample. Brain or hippocampal tissues were homogenized in ice-cold homogenization buffer (320 mM sucrose, 4 mM HEPES, pH 7.4, 2 mM EGTA, 50 mM NaF, 0.5 mM Na_3_VO_4_, 1 μM aprotinin, 1 μM leupeptin, and 1 μM pepstatin) using a glass homogenizer with 15–20 strokes. Homogenates were centrifuged for 15 min at 1,000 g at 4°C and the supernatants were centrifuged again for 15 min at 10,000 g at 4°C. Next, the resulting pellets were the crude synaptosomal fraction. The pellet was dissolved in hypo-osmotic buffer (2 mM EGTA, 50 mM NaF, 0.5 mM Na_3_VO_4_, 1 μM aprotinin, 1 μM leupeptin, and 1 μM pepstatin) by manually applying 5–7 strokes using a tissue grinder and followed by the addition of 1 M HEPES, pH 7.4. Then the hypo-osmotic synaptosomal fraction was rotated for 1 hour at 4°C. Hypo-osmotic synaptosomal fractions were centrifuged for 20 min at 25,000 g at 4°C and the pellets were snap frozen on dry ice. The pellets were resuspended in PSD preparation buffer (50 mM HEPES, pH 7.4, 2 mM EDTA, 2 mM EGTA, 50 mM NaF, 0.5 mM Na_3_VO_4_, 1 μM aprotinin, 1 μM leupeptin, and 1 μM pepstatin). To prepare the PSD fraction, 10% Triton X-100 was added to the resuspended pellets and they were incubated for 30 min at 4°C. Then, they were centrifuged for 20 min at 32,000 g (30,000 rpm in TLA100.2) at 4°C and the pellets were resuspended in the PSD preparation buffer to obtain PSD-1T fractions. The PSD-1T fractions were divided in half, and 10% Triton X-100 was added to one half, followed by 30 min incubation at 4°C and 15 min centrifugation at 200,000 g (75,000 rpm in TLA100.2) at 4°C. The pellets were resuspended in the PSD preparation buffer to obtain PSD-2T fractions. Using the bicinchoninic acid protein assay (Thermo Scientific Pierce), protein concentrations were measured for each sample.

**Morris water maze assay**

Mice were trained to find the hidden platform (10 cm diameter) in a white plastic tank with a diameter of 120 cm. Mice were given 3 trials per day with an inter-trial interval of 1 hour for six consecutive days. Behavior was recorded and the time taken to reach the platform (latency) was evaluated by Ethovision 3.1 program (Noldus).

**Open field assay**

The size of the open field box was 40 x 40 x 40 cm, and the center zone line was 10 cm from the edge. Mice were placed in the center of the chamber at the beginning of the assay, and mouse movements were recorded with a video camera for 30 min and analyzed by Ethovision 3.1 program (Noldus).

**Trace fear conditioning**

Standard delay fear conditioning tests were performed as previously described ([Chadman et al., 2008](#_lnxbz9); [Paylor et al., 2001](#_sqyw64)). Training and conditioning tests took place in two identical chambers (MED Associates) that were calibrated to deliver identical footshocks. Each chamber was 30 × 24 × 21 cm with a clear polycarbonate front wall, two stainless side walls, and a white opaque back wall. The bottom of the chamber consisted of a removable grid floor with a waste pan underneath. When placed in the chamber, the grid floor connected with a circuit board for delivery of scrambled electric shock. Each conditioning chamber was inside a sound-attenuating environmental chamber. A camera mounted on the front door of the environmental chamber recorded test sessions, which were later scored automatically, using VideoFreeze software (MED Associates). For the training session, each chamber was illuminated with a white house light. An olfactory cue was added by dabbing a drop of imitation banana flavoring solution (1:100 dilution in water) on the metal tray beneath the grid floor. The mouse was placed in the test chamber and allowed to explore freely for 2 min. A pure tone (5 kHz, 80 dB), which served as the conditioned stimulus (CS), was played for 30 sec. During the last 2 sec of the tone, a foot shock (0.5 mA) was delivered as the unconditioned stimulus (US). Each mouse received three CS–US pairings, separated by 90 sec intervals. After the last CS–US pairing, the mouse was left in the chamber for another 120 sec, during which freezing behavior was scored. The mouse was then returned to its home cage. Contextual conditioning was tested 24 hours later in the same chamber, with the same illumination and olfactory cue present but without foot shock. Each mouse was placed in the chamber for 5 min, in the absence of the CS and US, during which freezing was scored. The mouse was then returned to its home cage. Cued conditioning was conducted 48 hours after training. Contextual cues were altered by covering the grid floor with a smooth white plastic sheet, inserting a piece of black plastic sheet bent to form a vaulted ceiling, using near infrared light instead of white light, and dabbing vanilla instead of banana odor on the floor. The session consisted of a 3 min free exploration period, followed by 3 min of the identical CS tone (5 kHz, 80 dB). Freezing was scored during both 3 min segments. The mouse was then returned to its home cage. The chamber was thoroughly cleaned of odors between sessions, using 70% ethanol and water.

**References**

Chadman, K.K., Gong, S., Scattoni, M.L., Boltuck, S.E., Gandhy, S.U., Heintz, N., and Crawley, J.N. (2008). Minimal aberrant behavioral phenotypes of neuroligin-3 R451C knockin mice. Autism research : official journal of the International Society for Autism Research *1*, 147-158.

Paylor, R., Zhao, Y., Libbey, M., Westphal, H., and Crawley, J.N. (2001). Learning impairments and motor dysfunctions in adult Lhx5-deficient mice displaying hippocampal disorganization. Physiology & behavior *73*, 781-792.

Peca, J., Feliciano, C., Ting, J.T., Wang, W., Wells, M.F., Venkatraman, T.N., Lascola, C.D., Fu, Z., and Feng, G. (2011). Shank3 mutant mice display autistic-like behaviours and striatal dysfunction. Nature *472*, 437-442.
