# Supplementary material for: Loss of neuronal βPix isoforms impairs neuronal morphology in the hippocampus and causes behavioral defects
Source: Anim Cells Syst (Seoul). 2025 Jan 8;29(1):57–71. doi: 10.1080/19768354.2024.2448999 (PMC11722029; doi:10.1080/19768354.2024.2448999)
Supplement: Supplemental Material [file TACS_A_2448999_SM5991.zip › Supplementary Figures_R1.docx]

**Supplementary Figures**


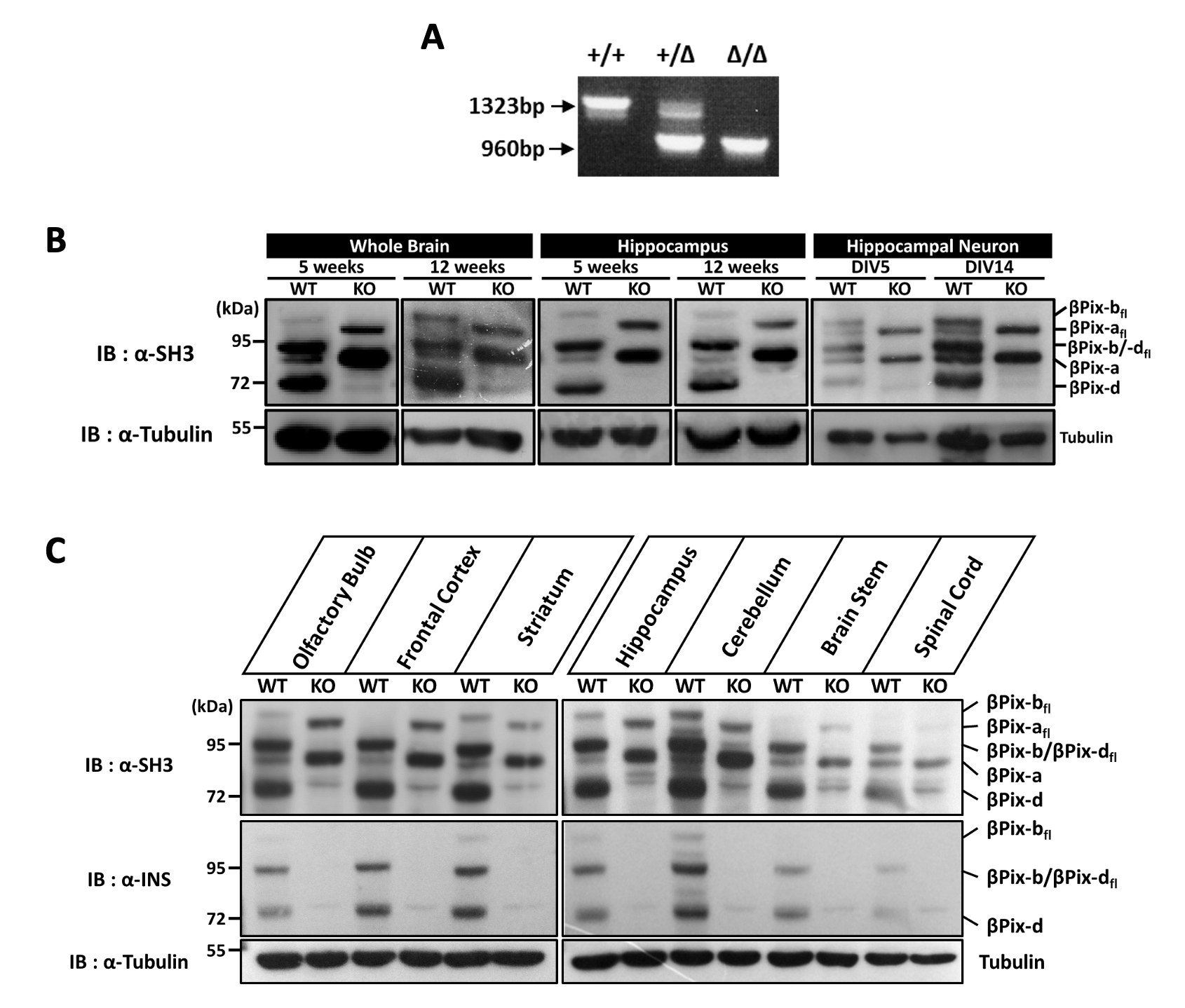


Supplementary Figure 1. Neuronal βPix isoform KO mice lack expression of βPix-b and βPix-d in brain regions, spinal cord, and cultured hippocampal neurons.

1. PCR genotyping for +/+, +/Δ, and Δ/Δ mice. Genotypes were determined by PCR of the tail DNA.
2. Expression patterns of βPix in the whole brain and hippocampus of 5- and 12-week-old mice, as well as DIV5 and DIV14 hippocampal neurons from WT and βPix-NIKO mice.
3. Expression patterns of βPix in the olfactory bulb, frontal cortex, striatum, hippocampus, cerebellum, brain stem, and spinal cord of 5-week-old WT and βPix-NIKO mice.


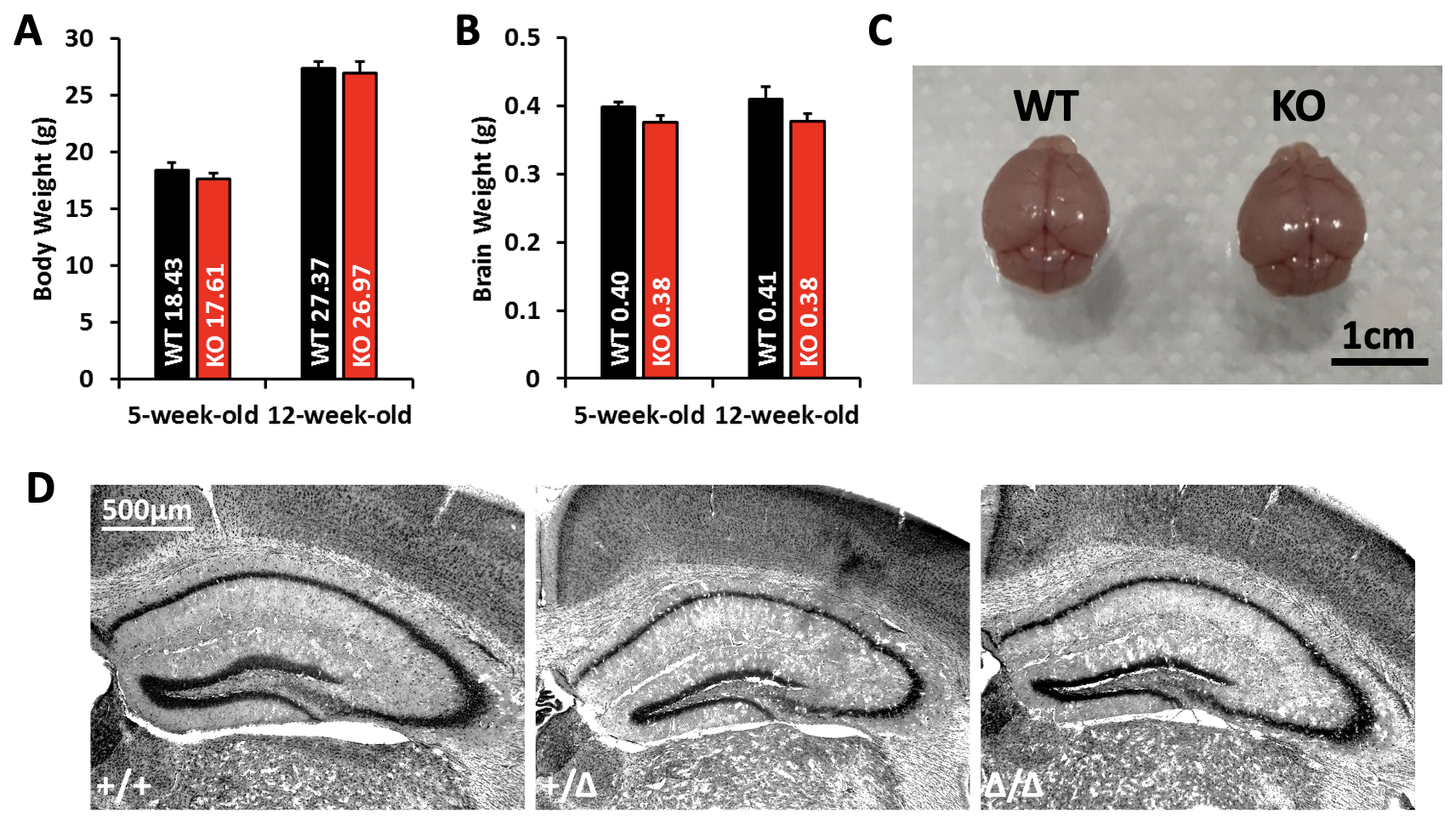


Supplementary Figure 2. Neuronal βPix isoform KO mice show normal body weight and brain morphology.

1. Body weights did not differ between WT and βPix-NIKO at 5 and 12 weeks of age.
2. Brain weight showed no differences between WT and βPix-NIKO mice at 5 and 12 weeks of age.
3. Twelve-week-old βPix-NIKO mice have normal brain size compared to littermate WT mice.
4. Images of Nissl-stained sagittal sections showed normal cytoarchitecture in the cortex and hippocampus of the 13-week-old male littermate mice.

n = 7 for 5-week-old and n = 6 for 12-week-old male littermate mice for (A) and (B).


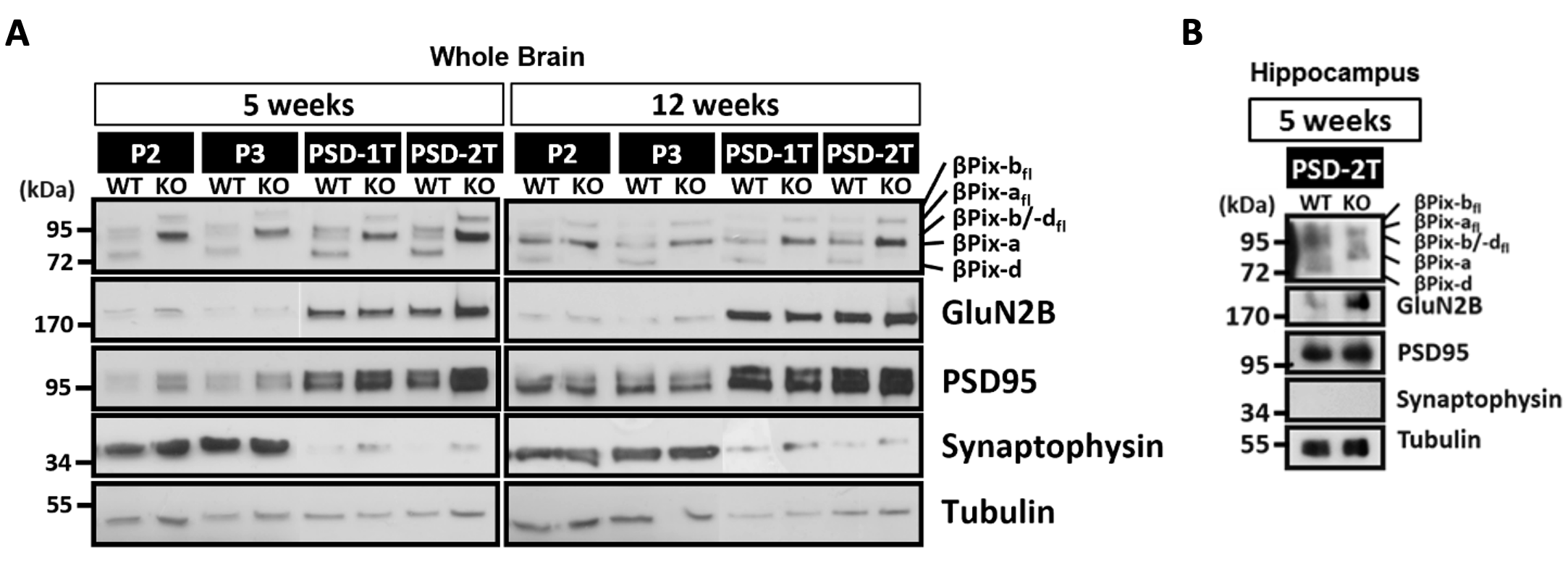


Supplementary Figure 3. Neuronal βPix isoform KO mice display increased GluN2B expression levels in hippocampal PSD fractions.

1. Expression of GluN2B protein in subcellular fractions of the whole brain from 5- and 12-week-old WT and βPix-NIKO mice. P2, crude synaptosomes; P3, light membrane fraction; PSD fractions, extracted with Triton X-100 once (PSD-1T) or twice (PSD-2T); An equal amount of 8 μg was loaded per fraction in immunoblot experiments. PSD-95 and synaptophysin were used as controls. GluN2B protein levels in the PSD-1T and 2T fractions of 5-week-old mice were increased in the absence of neuronal βPix isoforms, but this increase was not observed in 12-week-old mice.
2. Expression of GluN2B protein in hippocampal PSD fractions from 5-week-old mice. GluN2B protein was more enriched in βPix-NIKO PSD fractions compared to WT PSD fractions.


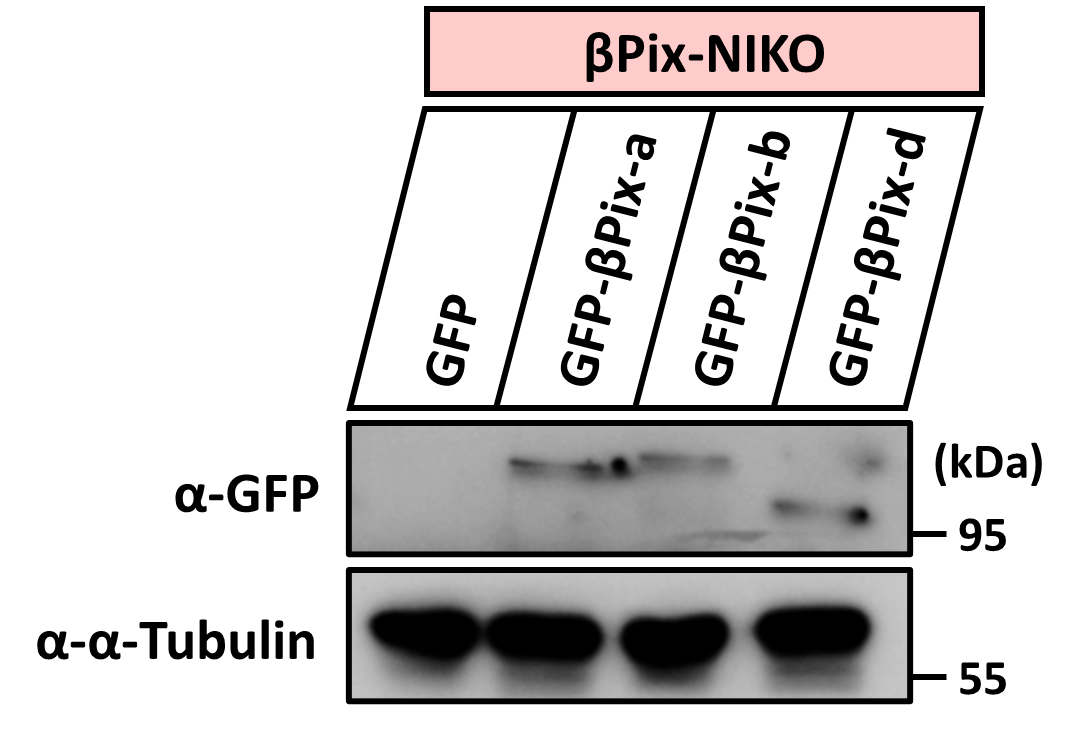


Supplementary Figure 4. Representative immunoblots showing levels of individually overexpressed βPix isoforms in βPix-NIKO hippocampal neurons. α-Tubulin was used as a loading control.


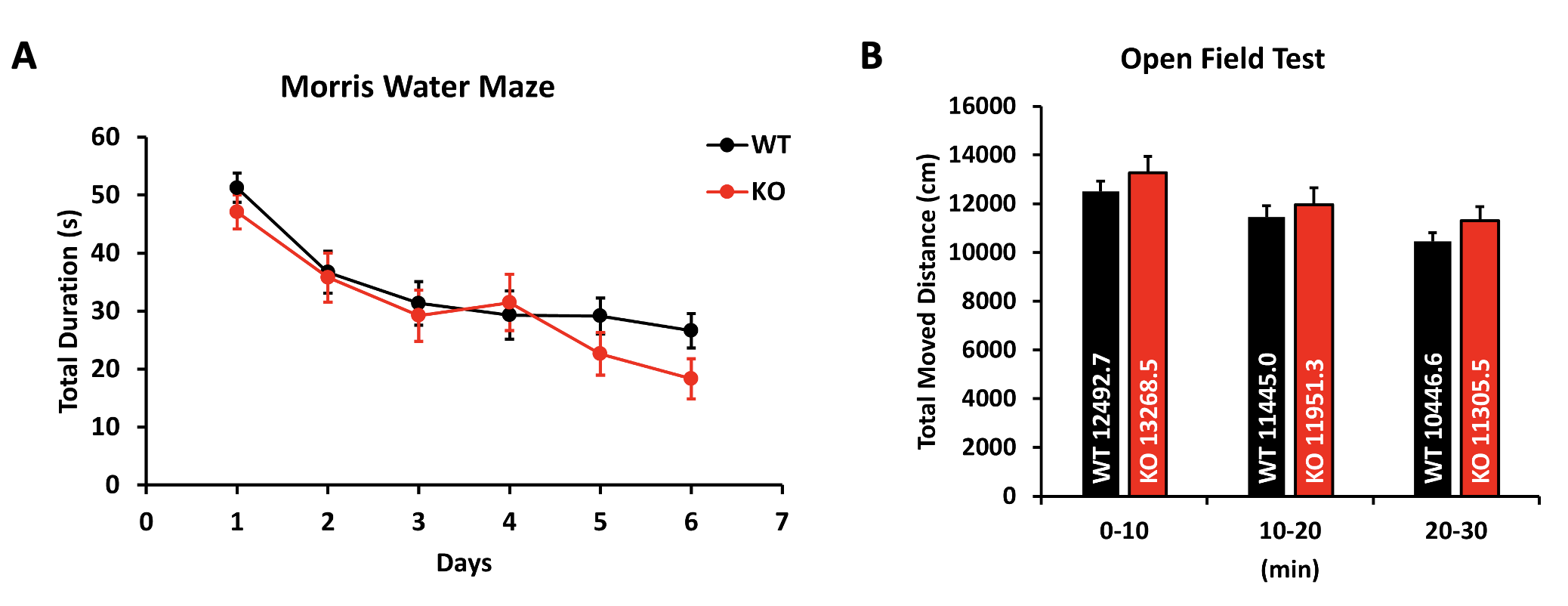


Supplementary Figure 5. Spatial learning and open field activity are normal in neuronal βPix isoform KO mice.

1. Spatial learning in the Morris water maze test was comparable between WT and βPix-NIKO mice. Total duration was defined as the time required to reach the submerged platform.
2. No genotype differences were found in total distance moved in the open field test.

n = 12 10–12-week-old male WT mice and n = 10 10–12-week-old male βPix-NIKO mice.


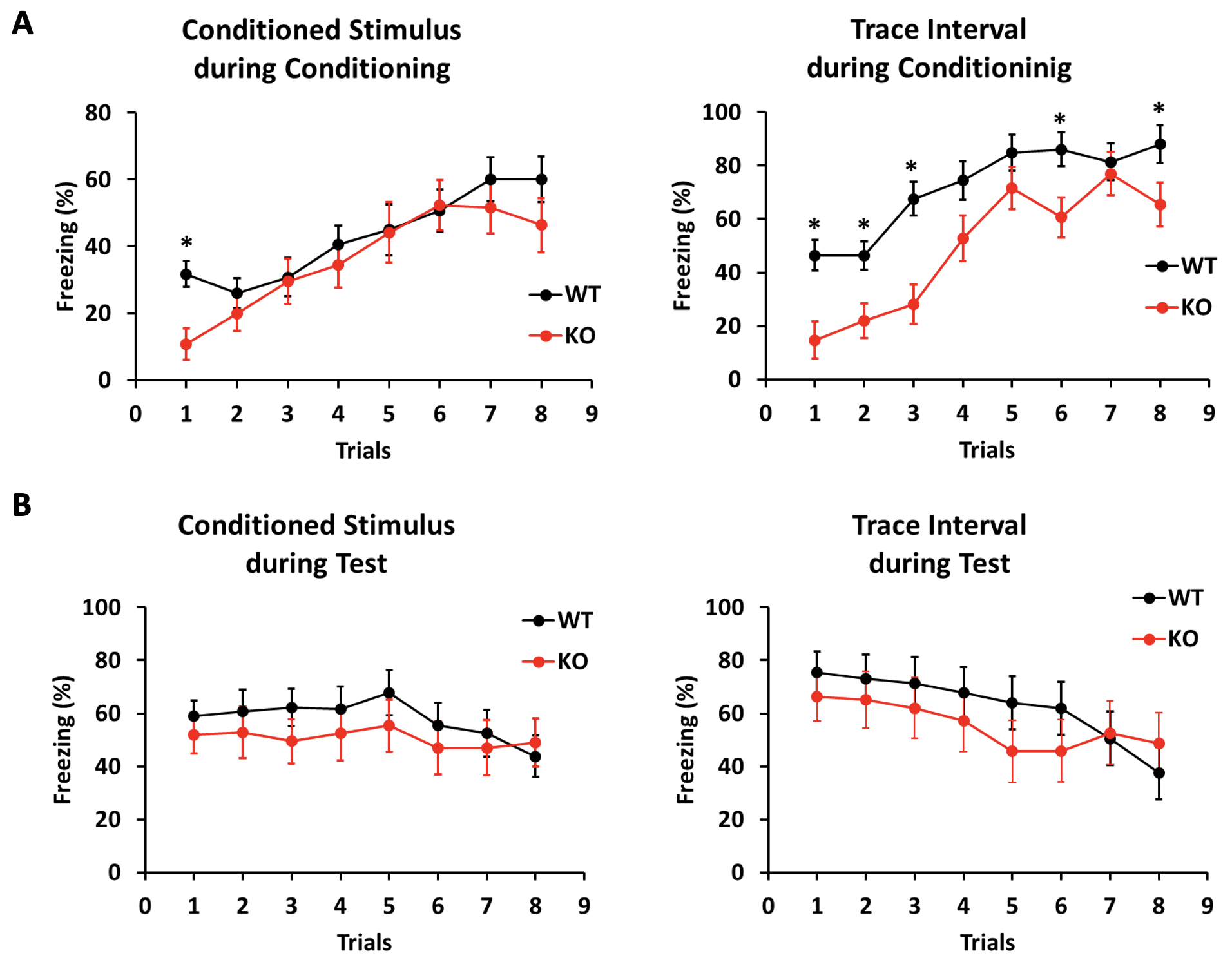


Supplementary Figure 6. Neuronal βPix isoform KO mice have low freezing levels during conditioning in trace fear conditioning test.

1. During conditioning, βPix-NIKO mice displayed lower levels of freezing behavior compared to WT mice.
2. During testing, no genotype differences were found in trace fear conditioning.

n = 12 10–12-week-old male WT mice and n = 10 10–12-week-old male βPix-NIKO mice. **P* < 0.05.
